# *Actinidia chinensis* Planch Root extract suppresses the growth and metastasis of hypopharyngeal carcinoma by inhibiting E2F Transcription Factor 1-mediated MNX1 antisense RNA 1

**DOI:** 10.1080/21655979.2022.2037226

**Published:** 2022-02-12

**Authors:** Yi Zheng, Lizhong Su, Jun Tan, Feilin Dong

**Affiliations:** Head and Neck & Otolaryngology Center, Plastic Surgery Center, Cancer Center, Department of Otolaryngology, Zhejiang Provincial People’s Hospital, Affiliated People’s Hospital, Hangzhou Medical College, Hangzhou, China

**Keywords:** *Actinidia chinensis* Planch Root extract, hypopharyngeal carcinoma, proliferation, metastasis, E2F1, MNX1-AS1

## Abstract

Increasing evidence has shown that traditional Chinese medicines and their bioactive components exert an anti-tumor effect, representing a novel treatment strategy. *Actinidia chinensis* Planch Root extracts (acRoots) have been reported to repress cancer cell proliferation and metastasis. The effect of acRoots on hypopharyngeal carcinoma progression was explored in this study. Firstly, data from MTT (3-(4,5-dimethylthiazol-2-yl)-2,5-diphenyltetrazolium bromide) and colony formation assays showed that incubation with accRoots reduced cell proliferation of hypopharyngeal carcinoma cells. Moreover, acRoots promoted the cell apoptosis of hypopharyngeal carcinoma. Secondly, cell migration and invasion of hypopharyngeal carcinoma cells were suppressed by acRoots. Thirdly, E2F1 (E2F Transcription Factor 1) and lncRNA MNX1-AS1 (MNX1 antisense RNA 1) were up-regulated in hypopharyngeal carcinoma tissues, and reduced in hypopharyngeal carcinoma cells post acRoots incubation. Overexpression of E2F1 attenuated acRoots-induced decrease in MNX1-AS1 in hypopharyngeal carcinoma cells. Lastly, administration with acRoots retarded in vivo hypopharyngeal carcinoma growth through down-regulation of E2F1-mediated MNX1-AS1. In conclusion, acRoots exerted tumor-suppressive role in hypopharyngeal carcinoma through inhibition of E2F1-mediated MNX1-AS1.

## Graphical abstract

**Figure:**
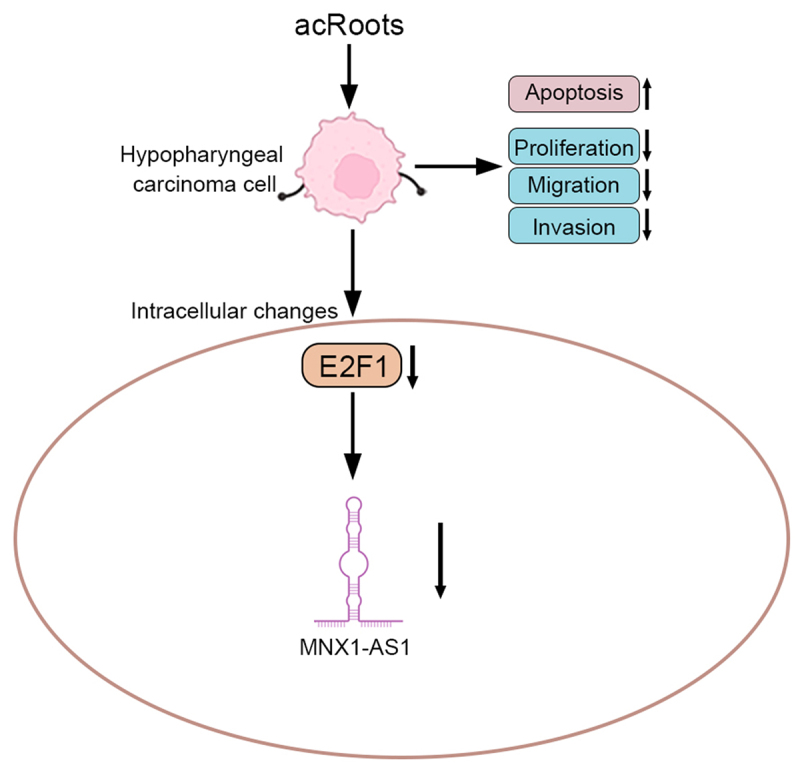

## Introduction

Hypopharyngeal carcinoma, accounting for 5%–15% of head and neck cancer cases, is originated from the mucosal epithelium of the hypopharynx [1]. Advances in the treatment strategies, such as radiotherapy, chemotherapy, and surgery, improve the 5-year survival rate of patients with hypopharyngeal carcinoma to 70% [2]. However, hypopharyngeal carcinoma is vulnerable to relapse, and the devoid of diagnostic biomarkers reduces the 5-year survival rate of patients with advanced hypopharyngeal carcinoma to 35% [3]. Therefore, novel prognostic biomarkers, as well as potential strategies, were urgently needed for the treatment of hypopharyngeal carcinoma.

*Actinidia chinensis* Planch, with the medicinal ingredients derived from the roots, is a traditional Chinese herbal medicine and exerts cardiovascular protective, antidiabetic, anti-inflammatory, antioxidant, hypolipemic, and immunoregulatory capacities [4]. *Actinidia chinensis* Planch was also widely used in the treatment of cancers [5]. For example, gastric cancer cell proliferation and migration were suppressed by *Actinidia chinensis* Planch [6]. AcRoots have been shown to suppress lung cancer proliferation through regulation of heat shock 70 kDa protein 6 [7]. Cell proliferation, metastasis, and epithelial–mesenchymal transition of hepatocellular carcinoma were also suppressed by acRoots [8]. However, the role of acRoots in the progression of hypopharyngeal carcinoma has rarely been reported.

AcRoots contain bioactive components, including quercetin, emodin, catechin, triterpenoids, flavonoids, and on on [9]. Quercetin [10] and emodin [11], the main components of acRoots, have been shown to exert antitumor activity. Both of quercetin [12] and emodin [13] reduced expression of transcriptional factor, E2F1, to suppress tumor progression. Moreover, E2F1 was also implicated in the tumorigenesis of nasopharyngeal carcinoma [14].

In this study, we hypothesized that acRoots might suppress progression of hypopharyngeal carcinoma through regulation of E2F1. The effects of acRoots on cell proliferation, apoptosis, migration, and invasion of hypopharyngeal carcinoma were investigated, and the specific mechanism was then evaluated to develop potential anti-cancer drug for the prevention of hypopharyngeal carcinoma.

## Materials and methods

### Tumor specimens

A total of 60 paired hypopharyngeal carcinoma and adjacent normal tissues were obtained from the patients at Zhejiang Provincial People’s Hospital, People’s Hospital of Hangzhou Medical College. Patients post preoperative radiotherapy or chemotherapy were excluded from this study according to previous study [15]. The written informed consents were acquired from all the patients. The study was approved by the Medical Ethics Committee of Zhejiang Provincial People’s Hospital and in accordance with the World Medical Association Declaration of Helsinki: Ethical principles for medical research involving human subjects.

### Cell culture

Human hypopharyngeal carcinoma cell, FaDu, was purchased from the American Type Culture Collection (Manassas, VA, USA) and cultured in Dulbecco’s modified Eagle’s medium with 10% fetal bovine serum (Gibco-BRL, Carlsbad, CA, USA) and penicillin-streptomycin at 37°C incubator according to previous study [15]. Human hypopharyngeal primary cells (HHPCs) were purchased from Celprogen Inc. (Carlsbad, CA, USA) and cultured in Human Hypopharyngeal Normal Cell Culture Media with Serum (Celprogen) according to previous study [16].

### Cell treatment and transfection

AcRoots were chopped and suspended in distilled water, according to previous study (*Actinidia chinensis* Planch root extract inhibits cholesterol metabolism in hepatocellular carcinoma through the up-regulation of PCSK9). Following heating at 100°C for 1 hour, the sediment was removed through a filter. The process was repeated twice, and the acRoots were equilibrated to 1 g/mL. FaDu cells were incubated with 50, 100, or 200 μg/mL extract for 72 hours before the functional assays. HHPCs were also incubated with 50, 100, 200, 400, or 800 μg/mL extract for 72 hours before the functional assays. FaDu with or without acRoots incubation were transfected with pcDNA-E2F1 (E2F1) or pcDNA vector (NC) by using Lipofectamine 2000 (Invitrogen, Carlsbad, CA, USA). Two days later, the cells were performed with functional assays. FaDu cells were also transfected with pcDNA-E2F1, shE2F1, pcDNA-MNX1-AS1, or shMNX1-AS1 by Lipofectamine 2000.

### Cell viability and proliferation assays

FaDu or HHPCs were seeded in a 96-well plate for 24, 48, or 72 hours and then treated with MTT solution (Dojindo, Tokyo, Japan) for 4 hours. Following incubation with dimethyl sulfoxide, absorbance at 570 nm was measured by Thermo Multiskan MK3 (Thermo Fisher Scientific Inc., Waltham, MA, USA) according to previous study [17]. For cell proliferation, FaDu cells were seeded in a 6-well plate for 10 days. Cells were fixed in methanol and then stained with crystal violet before photograph under light microscope (Olympus, Tokyo, Japan) according to previous study [18].

### Wound-healing assay

FaDu cells post indicated treatment were seeded into 6-well plates and then scratched by a pipette tip on the cell monolayers. The detached cells were washed and the wound width was calculated under a microscope (Olympus) 24 hours later according to previous study [19].

### Flow cytometry and transwell assays

FaDu cells post indicated treatment were harvested and suspended in the binding buffer of ApoDETECT Annexin V-FITC Kit (Thermo Fisher Scientific Inc.). After staining with fluorescently labeled Annexin V and propidium iodide (PI) (Thermo Fisher Scientific Inc.), the cells were performed with flow cytometry analyses by FACS flow cytometer (Attune, Life Technologies, Darmstadt, Germany) according to previous study [15]. For transwell assay, the FaDu cells post indicated treatment were suspended in a serum-free medium and plated into the upper chamber of the well (Corning, Tewksbury, MA, USA). Medium containing 15% fetal bovine serum was added into the lower chamber. Twenty-four hours later, cells in the lower chamber were stained with crystal violet and photographed under the microscope (Olympus). The upper chamber was precoated with Matrigel (BD Biosciences, Bedford, MA, USA) to investigate cell invasion according to previous study [15].

### Quantitative reverse transcription PCR (qRT-PCR)

RNAs were isolated from hypopharyngeal carcinoma tissues and FaDu cells using Trizol (Invitrogen), and then reverse-transcribed into cDNAs. SYBR Green Master (Roche, Mannheim, Germany) was used for the qRT-PCR analysis of E2F1 and MNX1-AS1 expression with following primers: E2F1 (forward: 5’-CACTTTCGGCCCTTTTGCTC-3’; reverse: 5’-GATTCCCCAGGCTCACCAAA-3’) and MNX1-AS1 (forward: 5’-GTGACTTCGCTGTGATGGA-3’; reverse: 5’-GGCCTCTATCTGTACCTTTATTCC-3’). GAPDH (forward: 5’-CTCTGCTCCTCCTGTTCGAC-3’; reverse: 5’-GCGCCCAATACGACCAAATC-3’) was used as endogenous control according to previous study [17].

### Western blot

Proteins extracted from hypopharyngeal carcinoma tissues and FaDu cells were separated by sodium dodecyl sulfate-polyacrylamide gel electrophoresis and then transferred onto polyvinylidene difluoride membrane. The membranes were blocked and probed with primary antibodies: anti-E2F1 (1:2000; Abcam, Cambridge, MA, USA) and anti-β-actin (1:2500; Abcam) antibodies. After incubation with the corresponding secondary antibody (1:5000; Abcam), the protein strips in the membrane were visualized by Colorimetric Western blotting Kit (Sigma-Aldrich) according to previous study [17].

### Animal model

Ethical approval of the animal experiment was obtained from the Experimental Animal Welfare Ethics Committee of Zhejiang Provincial People’s Hospital. Twenty BALB/c nude mice (4-week old, 20–22 g weight) were obtained from Shanghai Institute of Material Medicine (Shanghai, China), and then subcutaneously injected with FaDu cells to establish tumor model according to previous study [20]. The mice were divided into four groups: mice with 0, 75, 150, or 300 mg/kg acRoots (N = 5 in each group). For mice in each group, different concentrations of acRoots were intragastrically administered into the mice twice daily for 4 weeks. Mice were sacrificed, and the tumor tissues were collected. The tumor volumes were calculated, and the RNAs and proteins were extracted from the tumor tissues for functional analysis.

### Immunohistochemistry and TUNEL

Tumor tissues from the mice were fixed in 10% formalin and then embedded in paraffin. The tissues were sectioned into 4-µm-thick sections. After dewaxing and rehydration, the sections were incubated in 3% H_2_O_2_ and then immersed in Tris-EDTA buffer containing 0.05% Tween 20. Following incubation in 4% dry milk, the sections were incubated with anti-E2F1, or anti-Ki-67 (1:200; Abcam). After incubation with HRP-labeled secondary antibody, the slides were counterstained with hematoxylin and examined under the microscope (Olympus) according to previous study [20]. The tumor sections were incubated with TUNEL staining solution of TUNEL Assay Kit (Yanjinbio, Shanghai, China). The sections were also observed under the microscope (Olympus) according to a previous study [21].

### Statistical analysis

All the data were expressed as mean ± SD and analyzed by Student’s t-test or one-way analysis of variance. A *p* value of <0.05 was considered to be statistically significant.

## Results

### AcRoots suppressed cell proliferation of hypopharyngeal carcinoma

**Figure 1.**
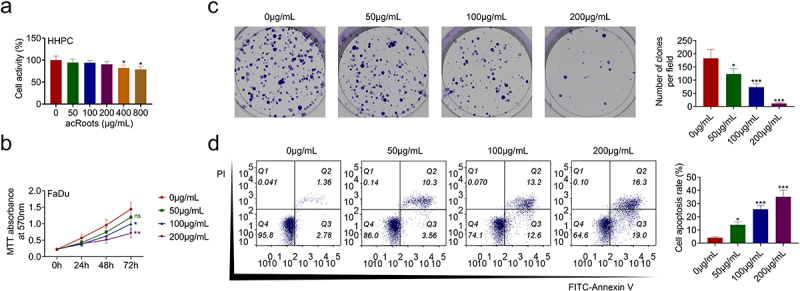
AcRoots suppressed cell proliferation of hypopharyngeal carcinoma.

To investigate the effect of acRoots on normal hypopharyngeal tissue cells, HHPCs were incubated with increasing concentrations of acRoots at 50, 100, 200, 400, or 800 μg/mL. MTT assay results showed that acRoots lower than 200 μg/mL did not affect the cell viability of HHPCs, while acRoots more than 400 μg/mL reduced the cell viability of HHPCs (Figure 1(a)). FaDu cells were also incubated with acRoots, and acRoots reduced cell viability of FaDu in a dosage-dependent way (Figure 1(b)). Cell proliferation of FaDu was also suppressed by acRoots (Figure 1(c)). Moreover, acRoots promoted the cell apoptosis of FaDu (Figure 1(d)), suggesting the anti-proliferative role of acRoots in hypopharyngeal carcinoma.

(a) Incubation with acRoots lower than 200 μg/mL did not affect the cell viability of HHPCs, while acRoots more than 400 μg/mL reduced the cell viability of HHPCs. (b) Incubation with acRoots reduced cell viability of FaDu in a dosage-dependent way. (c) Incubation with acRoots reduced cell proliferation of FaDu. (d) Incubation with acRoots promoted cell apoptosis of FaDu. *, **, *** vs. 0 μg/mL *Actinidia chinensis* Planch Root extract, *p* < 0.05, *p* < 0.01, *p* < 0.001.

### AcRoots suppressed cell migration invasion of hypopharyngeal carcinoma

**Figure 2.**
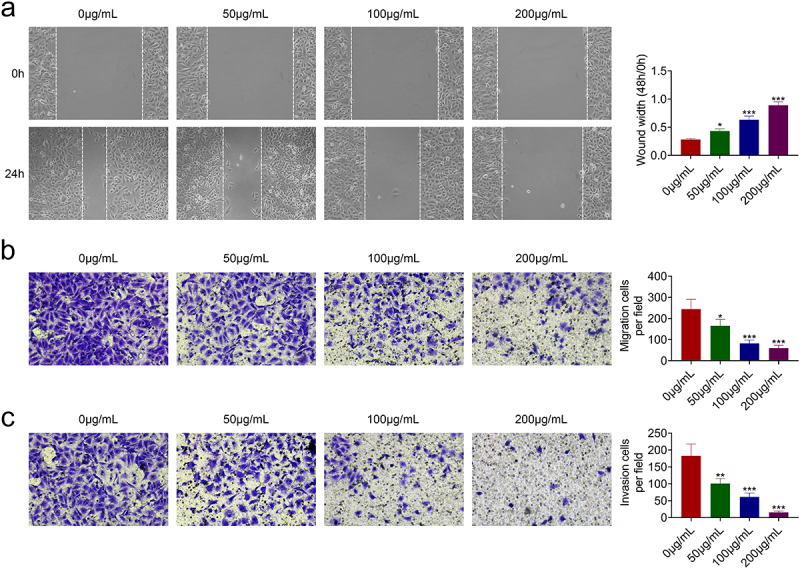
AcRoots suppressed cell migration invasion of hypopharyngeal carcinoma.

The effects of acRoots on cell migration and invasion of FaDu were then investigated. In addition to the anti-proliferative role, acRoots also suppressed cell migration of FaDu (Figure 2(a)). The number of migration (Figure 2(b)) and invasion (Figure 2(c)) cells in FaDu was reduced by acRoots in a dosage-dependent manner, demonstrating the anti-invasive role of acRoots in hypopharyngeal carcinoma.

(a) Incubation with acRoots reduced cell migration of FaDu. (b) Incubation with acRoots reduced the number of migrating cells in FaDu. (c) Incubation with acRoots reduced cell invasion of FaDu. *, **, *** vs. 0 μg/mL *Actinidia chinensis* Planch Root extract, *p* < 0.05, *p* < 0.01, *p* < 0.001.

### AcRoots reduced expression of E2F1 and MNX1-AS1

**Figure 3.**
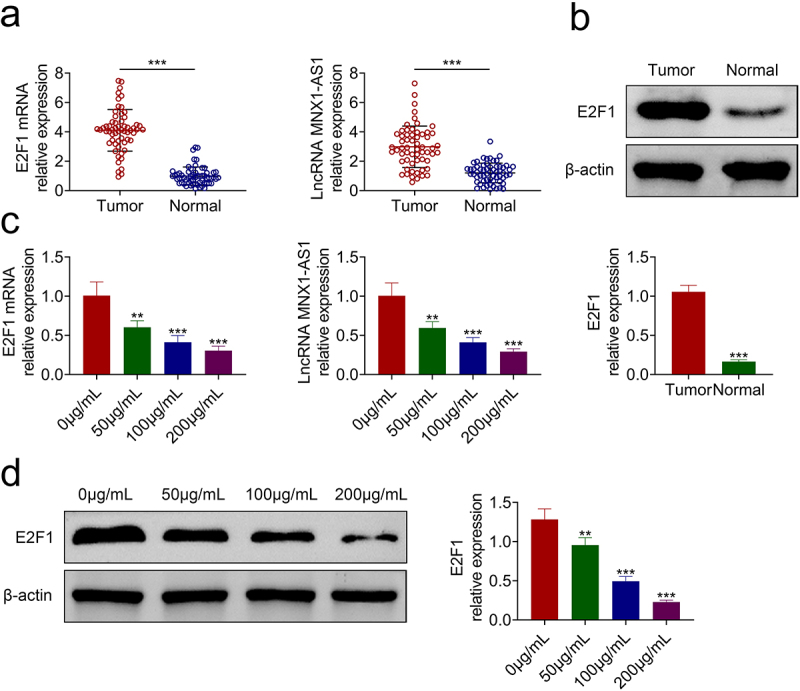
AcRoots reduced expression of E2F1 and MNX1-AS1.

To determine the mechanism involved in acRoots-mediated hypopharyngeal carcinoma progression, the expressions of E2F1 and MNX1-AS1 were detected, and the results showed that they were up-regulated in hypopharyngeal carcinoma tissues (Figure 3(a,b)). Incubation with acRoots decreased the expression of E2F1 and MNX1-AS1 (Figure 3(c,d)) in a dosage-dependent way, indicating that acRoots might regulate E2F1 and MNX1-AS1 to participate in the progression of hypopharyngeal carcinoma. FaDu was also transfected with pcDNA-E2F1, shE2F1 (Supplemental Figure S1(a)), pcDNA-MNX1-AS1, or shMNX1-AS1 (Supplemental Figure S2(a)) to investigate the effects of E2F1 or MNX1-AS1 on hypopharyngeal carcinoma. Overexpression of E2F1 (Supplemental Figure S1(b)) or MNX1-AS1 (Supplemental Figure S2(b)) promoted the cell proliferation of FaDu. The cell migration and invasion of FaDu cells were also enhanced by overexpression of E2F1 (Supplemental Figure S1(c,d)) or MNX1-AS1 (Supplemental Figure S2(c,d)). However, knockdown of E2F1 (Supplemental Figure S1(b-d)) or MNX1-AS1 (Supplemental Figure S2(b-d)) repressed the cell proliferation, migration, and invasion of FaDu.

(a) Expressions of E2F1 and MNX1-AS1 were up-regulated in hypopharyngeal carcinoma tissues. (b) Protein expression of E2F1 was up-regulated in hypopharyngeal carcinoma tissues. (c) Incubation with acRoots decreased expression of E2F1 and MNX1-AS1 in FaDu. (d) Incubation with acRoots decreased protein expression of E2F1 in FaDu. **, *** vs. 0 μg/mL acRoots or normal tissues, *p* < 0.01, *p* < 0.001.

### AcRoots reduced expression of MNX1-AS1 through inhibition of E2F1

**Figure 4.**
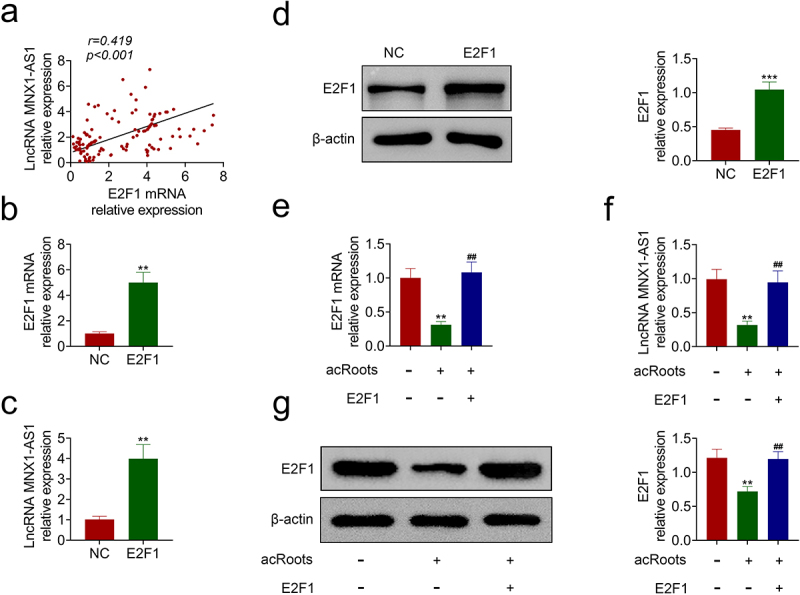
AcRoots reduced expression of MNX1-AS1 through inhibition of E2F1.

The effect of E2F1 on MNX1-AS1 was then investigated. Expression of E2F1 was positively associated with MNX1-AS1 in hypopharyngeal carcinoma (Figure 4(a)). FaDu cells were transfected with pcDNA-E2F1. Transfection with pcDNA-E2F1 promoted mRNA expression of E2F1 (Figure 4(b)) and enhanced the expression of MNX1-AS1 (Figure 4(c)). Protein expression of E2F1 was also up-regulated in FaDu cells transfected with pcDNA-E2F1 (Figure 4(d)). Moreover, the overexpression of E2F1 attenuated acRoots-induced decrease in E2F1 mRNA (Figure 4(e)), and the protein expression of MNX1-AS1 (Figure 4(f)) and E2F1 (Figure 4(g)) in FaDu, revealing that acRoots reduced expression of MNX1-AS1 through inhibition of E2F1.

(a) The expression of E2F1 was positively associated with MNX1-AS1 in hypopharyngeal carcinoma. (b) Transfection with pcDNA-E2F1 promoted mRNA expression of E2F1 in FaDu. (c) Transfection with pcDNA-E2F1 promoted expression of MNX1-AS1 in FaDu. (d) Transfection with pcDNA-E2F1 promoted protein expression of E2F1 in FaDu. (e) Overexpression of E2F1 attenuated *Actinidia chinensis* Planch Root extract-induced decrease of E2F1 mRNA. (f) Overexpression of E2F1 attenuated *Actinidia chinensis* Planch Root extract-induced decrease of MNX1-AS1. (g) Overexpression of E2F1 attenuated *Actinidia chinensis* Planch Root extract-induced decrease of E2F1 protein. **, *** vs. NC, *p* < 0.01, *p* < 0.001. ## vs. acRoots, *p* < 0.01.

### AcRoots suppressed cell proliferation and metastasis of hypopharyngeal carcinoma through regulation of E2F1

**Figure 5.**
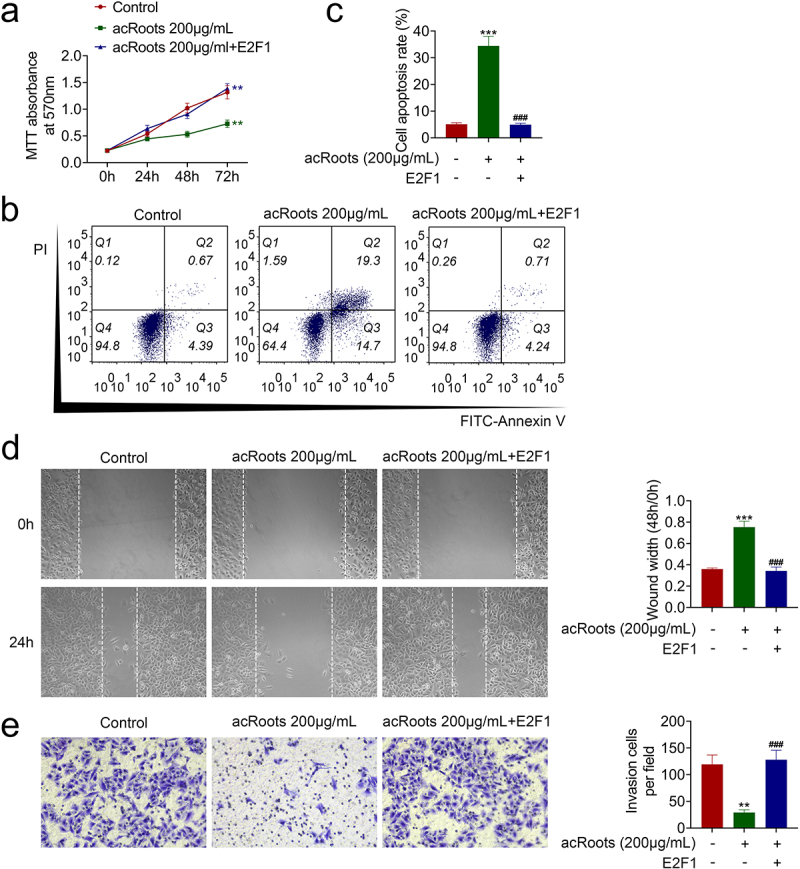
AcRoots suppressed cell proliferation and metastasis of hypopharyngeal carcinoma through regulation of E2F1.

FaDu with acRoots incubation were transfected with pcDNA-E2F1 to detect the effect of acRoots/E2F1 on hypopharyngeal carcinoma growth. The overexpression of E2F1 attenuated the acRoots-induced decrease in cell viability of FaDu (Figure 5(a)). AcRoots-induced increase in cell apoptosis in FaDu was reduced by overexpression of E2F1 (Figure 5(b,c)). The overexpression of E2F1 counteracted with the suppressive effects of acRoots on cell migration (Figure 5(d)) and invasion (Figure 5(e)) in FaDu, revealing that acRoots suppressed hypopharyngeal carcinoma progression through down-regulation of E2F1.

(a) Overexpression of E2F1 attenuated acRoots-induced decrease in cell viability in FaDu. (b) Overexpression of E2F1 attenuated acRoots-induced increase in cell apoptosis in FaDu. (c) The relative apoptosis rate in acRoots-induced FaDu with or without pcDNA-E2F1 transfection. (d) Overexpression of E2F1 attenuated acRoots-induced decrease in cell migration in FaDu. (e) Overexpression of E2F1 attenuated acRoots-induced decrease in cell invasion in FaDu. **, *** vs. NC, *p* < 0.01, *p* < 0.001. ### vs. acRoots, *p* < 0.001.

### AcRoots suppressed in vivo hypopharyngeal carcinoma growth

**Figure 6.**
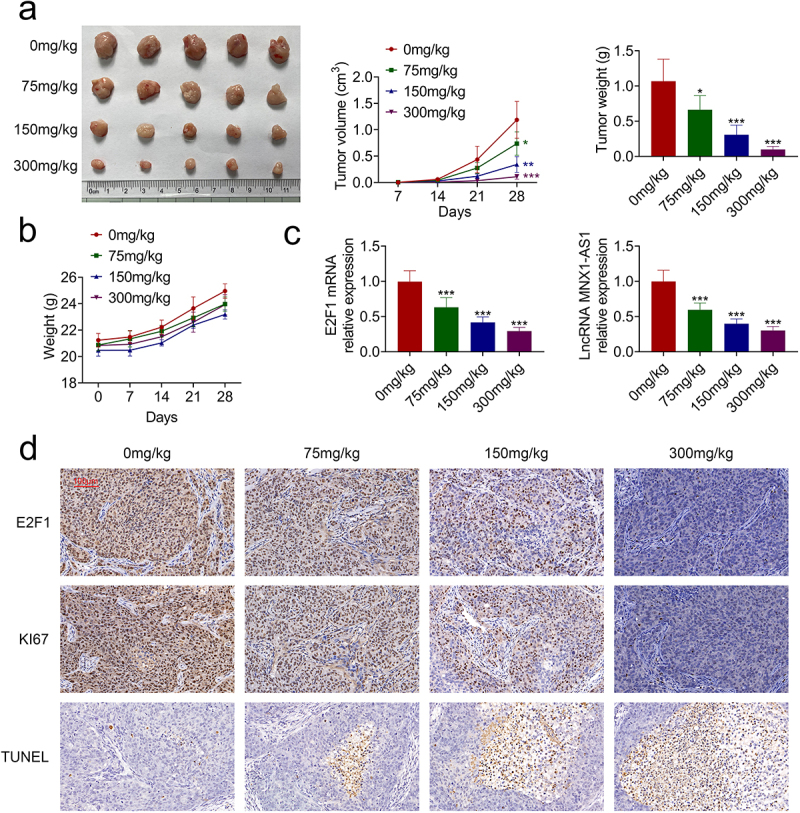
AcRoots suppressed *in vivo* hypopharyngeal carcinoma growth.

*In vivo* effect of acRoots on hypopharyngeal carcinoma growth was then evaluated. Intragastrically administered acRoots repressed tumor growth of hypopharyngeal carcinoma with reduced tumor volume and weight in a dosage-dependent way (Figure 6(a)). The body weight changes of the experimental mice are shown in Figure 6(b), suggesting no obvious toxicity of acRoots. The expressions of E2F1 and MNX1-AS1 in the tumor tissues were also decreased by acRoots (Figure 6(c)). Immunohistochemical analysis indicated down-regulation of E2F1 and Ki67 in the tumor tissues (Figure 6(d)). Moreover, incubation with acRoots enhanced the number of TUNEL-positive cells in the tumor tissues (Figure 6(d)), which further confirmed the tumor-suppressive role of acRoots in hypopharyngeal carcinoma.

(a) Intragastrically administered acRoots repressed tumor growth of hypopharyngeal carcinoma with reduced tumor volume and weight. (b) No sudden weight loss of mice was observed in the acRoots group, and there was no significant difference in weight between different groups. (c) Intragastrically administered acRoots reduced the expression of E2F1 and MNX1-AS1. (d) Immunohistochemical analysis indicated down-regulation of E2F1 and Ki67 in the tumor tissues. Incubation with acRoots increased the number of TUNEL-positive cells in the tumor tissues. *, *** vs. 0 mg/mL acRoots, *p* < 0.05, *p* < 0.001.

## Discussion

Increasing evidence has pointed out that various Chinese herbal medicines and their bioactive components exert anti-tumor effects, representing a novel tumor therapeutic strategy [22]. In the treatment of head and neck cancer, the natural compounds isolated from the Chinese herbal medicines induced growth inhibition of cancer cells and improved resistance to chemotherapy [23]. Considering the anti-tumor effect of the acRoots, the effect and mechanism of acRoots on progression of hypopharyngeal carcinoma were then investigated in this study.

AcRoots in this study suppressed cell proliferation of hypopharyngeal carcinoma and promoted cell apoptosis. Moreover, the cell migration and invasion of hypopharyngeal carcinoma were also repressed by acRoots. Additionally, *in vivo* administration of acRoots also suppressed tumor growth in hypopharyngeal carcinoma, suggesting the anti-tumor effect of acRoots on hypopharyngeal carcinoma.

Chinese herbal medicine or its derived compounds regulated T cell differentiation [24], and genes involved in proliferation, metastasis, angiogenesis, or apoptosis [25] to participate in the tumor progression. Transcription factors were also involved in the regulation of Chinese herbal medicine or its derived compounds in tumorigenesis [26]. Prostaglandin E receptor 3 was identified as a regulator involved in the response of hepatocellular carcinoma to acRoots [27]. AcRoots also suppressed DLX2/TARBP2/JNK/AKT pathway to inhibit hepatocellular carcinoma cell proliferation and metastasis [28]. Our results showed that transcriptional factor, E2F1, was reduced in hypopharyngeal carcinoma tissues and cells post acRoots incubation. Moreover, the overexpression of E2F1 attenuated acRoots-induced increase in cell apoptosis in FaDu, and decrease in cell viability, migration, and invasion. Therefore, acRoots exerted anti-tumor effect against hypopharyngeal carcinoma through down-regulation of E2F1.

E2F1 has been shown to promote the progression of colon adenocarcinoma through the up-regulation of lncRNA MNX1-AS1, and E2F1 can bind to the promoter region of MNX1-AS1 [29]. MNX1-AS1 functions as an oncogene, to promote epithelial–mesenchymal transition of breast cancer [30], cell proliferation, and metastasis of esophageal squamous cell carcinoma [31], bladder cancer [32], cervical cancer [33], and intrahepatic cholangiocarcinoma [34]. Knockdown of MNX1-AS1 suppressed ovarian cancer cell migration and proliferation [35]. Moreover, MNX1-AS1 also promoted cell migration and growth of laryngeal squamous cell carcinoma [36]. Here, MNX1-AS1 was up-regulated in the hypopharyngeal carcinoma tissues, while down-regulated in hypopharyngeal carcinoma cells post acRoots incubation. Overexpression of E2F1 attenuated *Actinidia chinensis* Planch root extract-induced decrease in MNX1-AS1 expression in hypopharyngeal carcinoma cells. These results showed that acRoots might suppress hypopharyngeal carcinoma progression through reduction of E2F1-mediated MNX1-AS1 expression. However, the effects of E2F1/MNX1-AS1 on the progression of hypopharyngeal carcinoma post acRoots incubation should be investigated in further research.

## Conclusion

In summary, acRoots suppressed cell proliferation and metastasis of hypopharyngeal carcinoma. E2F1-mediated MNX1-AS1 expression was implicated in the anti-tumor effect of acRoots on hypopharyngeal carcinoma. The results of this study might provide a novel therapeutic strategy for the prevention of hypopharyngeal carcinoma growth and metastasis.

## Supplementary Material

Supplemental Material

## Data Availability

All data generated or analyzed during this study are included in this published article.

## Highlights

1. *Actinidia chinensis* Planch Root extract suppressed cell proliferation of hypopharyngeal carcinoma.
2. *Actinidia chinensis* Planch Root extract suppressed cell migration invasion of hypopharyngeal carcinoma.
3. *Actinidia chinensis* Planch Root extract reduced expression of MNX1-AS1 through inhibition of E2F1.
4. *Actinidia chinensis* Planch Root extract suppressed in vivo hypopharyngeal carcinoma growth.
