# Supplementary figures and images for: Actinidia chinensis Planch Root extract suppresses the growth and metastasis of hypopharyngeal carcinoma by inhibiting E2F Transcription Factor 1-mediated MNX1 antisense RNA 1
Source: Bioengineered. 2022 Feb 12;13(3):4911–22. doi: 10.1080/21655979.2022.2037226 (PMC8973797; doi:10.1080/21655979.2022.2037226)

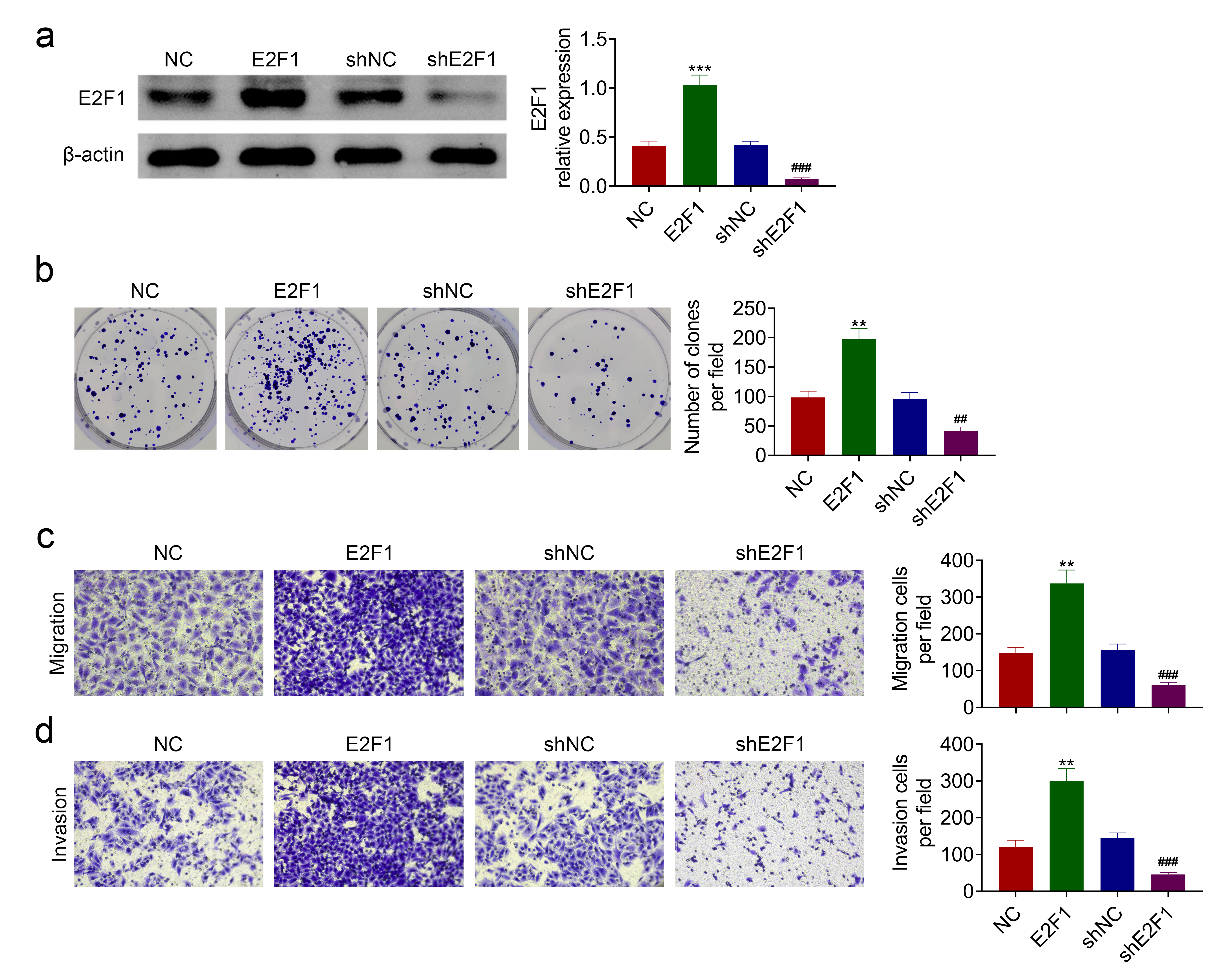

Supplement: Supplemental Material [file KBIE_A_2037226_SM2811.zip › supplementary/supplement FIG 1.jpg]

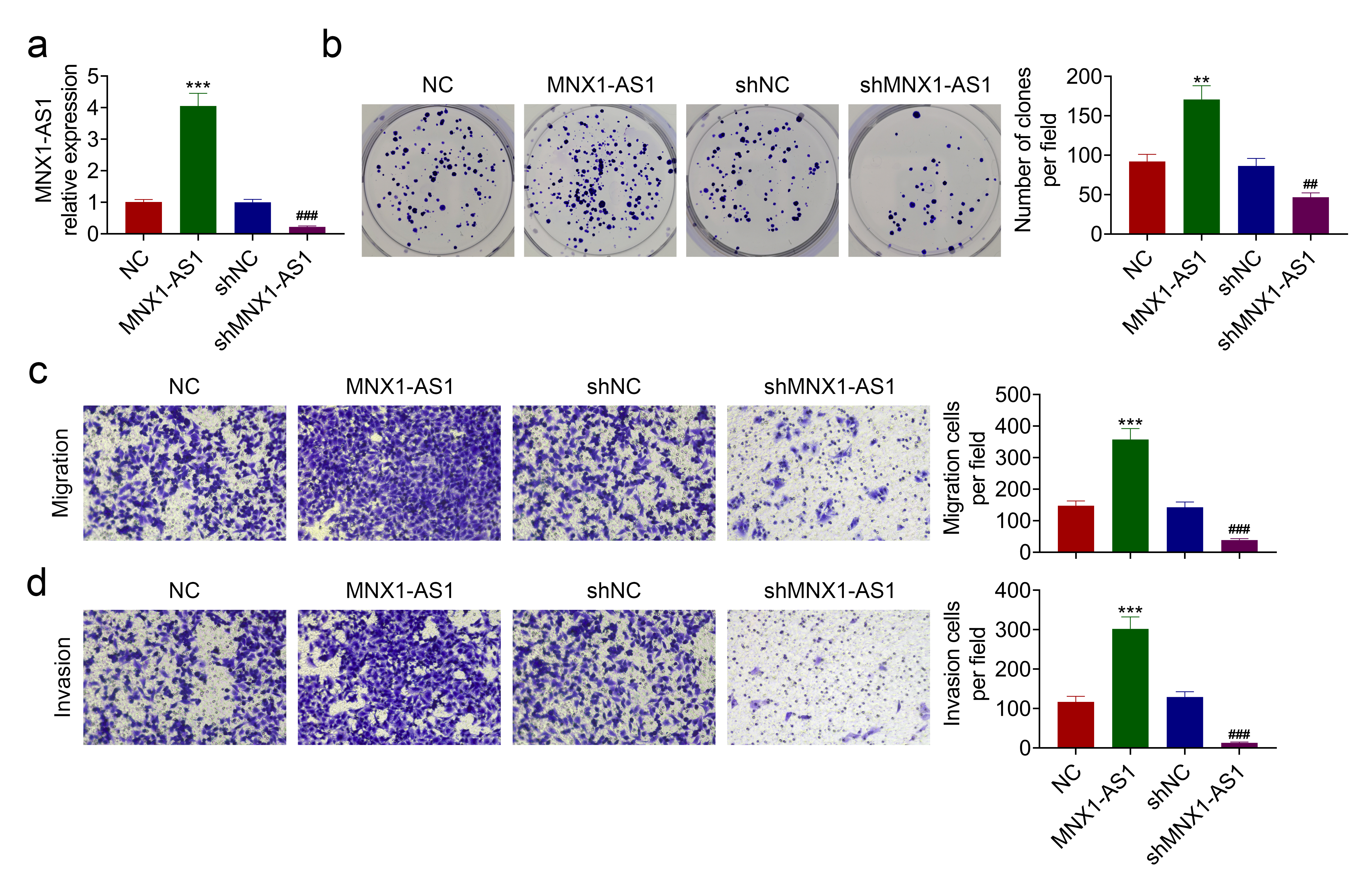

Supplement: Supplemental Material [file KBIE_A_2037226_SM2811.zip › supplementary/supplement FIG 2.jpg]
